# Supplementary material for: Surface conjugation of antibodies improves nanoparticle uptake in bronchial epithelial cells
Source: PLoS One. 2022 Apr 6;17(4):e0266218. doi: 10.1371/journal.pone.0266218 (PMC8986008; doi:10.1371/journal.pone.0266218)
Supplement: Attachment — Submitted filename: Response to Reviewers.docx [file pone.0266218.s001.docx]

Editor Comments:

1. “… funding information should not appear in the Acknowledgments section or

other areas of your manuscript.”

*The Acknowledgements section has been removed.*

2. “We note that you have included the phrase “data not shown” in your manuscript”

*The sentence and the phrase “data not shown” have been removed from the manuscript”*

3. “Please include your full ethics statement in the ‘Methods’ section of your manuscript file.”

*These studies were performed on cell lines with synthesized particles there is no human experiments or animal experiments. Therefore, there is no IRB or animal protocol needed for this work.*

Our current Funding Statement is accurate:

[DHS STITEL17GO Cystic Fibrosis Foundation
<https://www.cff.org/>
The funders had no role in study design, data collection and analysis, decision to publish, or preparation of the manuscript.

WMS UG3- HL147352 National Institutes of Health
<https://www.nih.gov/institutes-nih>
The funders had no role in study design, data collection and analysis, decision to publish, or preparation of the manuscript]

4. ” PLOS requires an ORCID iD for the corresponding author in Editorial Manager…”

The ORCID iD for David Stitelman is below and was uploaded into the Editorial Manager.

[https://orcid.org/0000-0001-8067-5830](https://nam12.safelinks.protection.outlook.com/?url=https%3A%2F%2Forcid.org%2F0000-0001-8067-5830%3Flang%3Den&data=04%7C01%7Cdavid.stitelman%40yale.edu%7C7b0ba6792b854972d16308da05536352%7Cdd8cbebb21394df8b4114e3e87abeb5c%7C0%7C0%7C637828152954593350%7CUnknown%7CTWFpbGZsb3d8eyJWIjoiMC4wLjAwMDAiLCJQIjoiV2luMzIiLCJBTiI6Ik1haWwiLCJXVCI6Mn0%3D%7C3000&sdata=nHKsC%2FSwYDJHyssrYkmFF4WWeFxV%2FtPr62VhacU5tNc%3D&reserved=0)

Reviewer Comments:

Major points:

1. Based on the description of the conjugated nanoparticle treatment on page 9, the authors analyzed the mean fluorescence intensity of “cells that took up fluorescent loaded NPs”. How was the percentage of cells that took up the NPs?

*Our collaborators have classically looked at mean fluorescence intensity to see if more particles were going into each cell. We had not analyzed the data looking at cell number with particle uptake previously and were impressed by these results. Upon review of the flow cytometry data, we see that at our low dose, the conjugated particles enter over 50% of cells compared to less than a percent of cells (60 fold increase) and with the higher dose, conjugated particles enter about 98% of cells compared to 14% of cells (7 fold increase). This has been added to the figures, results and discussion.*

2. The authors should consider including images for the CFBE cells that internalized NPs for the data shown in Figure 3C.

*We have no more of these particles and do not have these images. We feel the flow cytometry data is quantitative and the flow cytometry analysis has been improved.*

3. Individual data points should be presented in Figure 3A and 3C.

*We have removed figure 3A to make room for what are now figure 3B and 3C. We have changed the graphs from bar graphs to graphs of individual data points.*

4. The authors used CFBE cells, which carry the F508 deletion mutation, as the in vitro model in this study, and described the significance of improving the approaches used for molecular therapy for cystic fibrosis to a great extent. Have the authors observed any effect(s) using the NPs loaded with gene editing agents? Will the improved NP uptake benefit the outcomes/functional readout of gene editing in the CFBE cells?

*The conjugation technique that we have used for this project results in the antibody to be oriented in random direction so the binding domain may be facing away from the particle (ideal) but more often aimed in the wrong direction. We have developed a new method of antibody conjugation that results in the antibodies aimed in the ideal direction and this new generation of particles enters cells more efficiently than the method described in this manuscript. The editing reagents are expensive and in limited supply and we will not be using them in antibody conjugated particles synthesized in the manner described in this paper. We feel that this demonstration of increased particle uptake using antibody conjugation is important and studies of intracellular release and therapeutic efficacy are a necessary next step.*

Minor points:

1. It might help the readers if the rationale of target selection on page 6 is explained at the beginning of the Results session.

*A sentence describing the rationale was added to the beginning of the result section on page 9*.

1. Labeling and units for the y-axes are missing in Figure 2 and Figure 3C.

*The figures are now labeled accurately.*

2. The font used for “Greek letter mu (µ)” symbols needs to be standard and unified throughout the manuscript.
3. All “Greek letter mu” were changed to µ.
4. Page 12. Line 3, what is “DiO” short for?
